# Differential miRNA-Gene Expression in M Cells in Response to Crohn’s Disease-Associated AIEC

**DOI:** 10.3390/microorganisms8081205

**Published:** 2020-08-07

**Authors:** Anaïs Larabi, Laurène Salesse, Charlotte Cordonnier, Lucie Etienne-Mesmin, Nicolas Barnich, Guillaume Dalmasso, Hang Thi Thu Nguyen

**Affiliations:** 1 M2iSH, UMR 1071 Inserm, Université Clermont Auvergne, INRAE USC 2018, CRNH, 63001 Clermont-Ferrand, France; anais.larabi@gmail.com (A.L.); laurene.salesse@uca.fr (L.S.); cordonnier.charlotte@wanadoo.fr (C.C.); nicolas.barnich@uca.fr (N.B.); guillaume.dalmasso@uca.fr (G.D.); 2 MEDIS, INRAE, Université Clermont Auvergne, 63001 Clermont-Ferrand, France; lucie.etienne-mesmin@uca.fr

**Keywords:** Crohn disease, Adherent-invasive *E. coli*, M cells, MicroRNAs, Transcriptomic analysis

## Abstract

Adherent-invasive *Escherichia coli* (AIEC), which abnormally colonize the ileal mucosa of Crohn’s disease (CD) patients, are able to invade intestinal epithelial cells (IECs) and translocate through M cells overlying Peyer’s patches. The levels of microRNA (miRNA) and gene expression in IECs and M cells upon AIEC infection have not been investigated. Here, we used human intestinal epithelial Caco-2 monolayers and an in vitro M-cell model of AIEC translocation to analyze comprehensive miRNA and gene profiling under basal condition and upon infection with the reference AIEC LF82 strain. Our results showed that AIEC LF82 translocated through M cells but not Caco-2 monolayers. Both differential gene expression and miRNA profile in M cells compared to Caco-2 cells were obtained. In addition, AIEC infection induces changes in gene and miRNA profiles in both Caco-2 and M cells. In silico analysis showed that certain genes dysregulated upon AIEC infection were potential targets of AIEC-dysregulated miRNAs, suggesting a miRNA-mediated regulation of gene expression during AIEC infection in Caco-2, as well as M cells. This study facilitates the discovery of M cell-specific and AIEC response-specific gene-miRNA signature and enhances the molecular understanding of M cell biology under basal condition and in response to infection with CD-associated AIEC.

## 1. Introduction

Crohn’s disease (CD) is an inflammatory bowel disease (IBD) with a multifactorial etiology, involving a complex interaction between environmental and microbial factors in a genetically susceptible individual, leading to abnormal immune responses to gut microbiota [1]. An intestinal dysbiosis, characterized by a decrease in beneficial bacteria such as members of the Firmicutes phylum and an increase in potentially pathogenic bacteria such as members of Enterobacteriaceae family, has been described in patients with CD [2]. Particularly, adherent-invasive *E. coli* (AIEC) strains, found with higher prevalence in the ileal mucosa of CD patients compared to healthy subjects, have been involved in CD pathogenesis [3]. AIEC strains have the ability to adhere to and to invade intestinal epithelial cells (IECs) [4], to translocate through M cells overlying Peyer’s patches (PPs) via long polar fimbriae (LPF) [5], to survive and replicate within macrophages [6] with increased replication in macrophages from CD patients compared to healthy controls [7], and to trigger a pro-inflammatory response in host cells [6,8,9,10]. Furthermore, AIEC are able to colonize the gut of genetically susceptible murine models, thus inducing gut dysbiosis and intestinal inflammation [11,12,13]. Although autophagy is induced in host cells to restrain AIEC replication [9,10,14], these bacteria can subvert autophagy via the modulation of host miRNAs or SUMOylation consequently leading to increased AIEC intracellular replication and AIEC-induced inflammation [8,15,16]. We also showed that upon AIEC infection, IECs and macrophages secrete exosomes, extracellular vesicles of 30 to 100 nm, that can consequently increase pro-inflammatory cytokine production and AIEC replication in exosome-receiving cells [17,18].

MicroRNA may play a role in the pathogenesis of IBD, and several studies have reported a dysregulated expression of miRNAs in both serum and tissue samples of IBD patients [19]. Although the biological role and mRNA targets of most of these miRNAs remain to be identified, the function and impact of some of them have been discovered. In CD, a single nucleotide polymorphism (SNP) located in the gene encoding immunity-related GTPase M (IRGM) was reported to alter the binding site of miR-196, resulting in the deregulation of IRGM expression and the subsequent impaired autophagy to control AIEC intracellular replication [20]. AIEC bacteria are also able to counteract the autophagy response of infected cells by modulating the levels of several CD-associated miRNAs that target autophagy-related genes, thus enhancing AIEC intracellular replication and AIEC-induced inflammation [8]. Therefore, miRNAs have emerged as key players in the regulation of intracellular processes in response to AIEC infection.

In CD, initial mucosal lesions commonly occur in the distal ileum and colonic lymphoid follicles on the site of the follicle-associated epithelium (FAE) [21,22], an epithelium composed of specialized IECs that form the junction between the lumen and the intestinal lymphoid system [23]. The major roles of FAE, which are undertaken by microfold or membranous cells (M cells) located in the FAE, are to uptake macromolecules, antigens, and microorganisms from the intestinal lumen and deliver them to the underlying lymphoid tissue to initiate subsequent mucosal immune responses [23]. In particular, M cells recognize luminal antigens via cell-surface receptors and deliver them, via the transcytosis, to mononuclear phagocytes such as dendritic cells (DCs) and macrophages, and B cells localized in the PPs to trigger antigen-specific immune responses such as antigen-specific secretory IgA production. However, various infectious agents, such as *Salmonella* Typhimurium and *Shigella* spp., exploit M cells as a portal for invasion [23]. Thus, M cells have a dual character: they have a key role in maintaining gut immune homeostasis and a mutual relationship with the gut microbiota, and represent a gate for pathogens to establish an infection.

While ex vivo studies have reported a defective mucosal barrier of Peyer’s patches to bacteria in CD patients [24,25], other studies have revealed that AIEC can invade their host and disseminate via the colonization of both human and murine PPs [5,26,27,28] and translocation across human PPs and more specifically M cells [5,26,27,28,29]. Thus, an increased amount of AIEC at M cells may play a role in CD development. While M cells seem to be involved in the colonization and dissemination of AIEC in CD, the cellular responses of M cells to AIEC infection remain to be clarified.

By identifying dysregulated miRNAs and genes in the human intestinal epithelial cell line Caco-2 and in a human M-cell model upon AIEC infection, we aimed to reveal the potential cellular pathways involved in the response of these cells to AIEC bacterial invasion. Characterization of the changes in miRNA-gene interaction in IECs and M cells upon AIEC infection may help to understand the molecular mechanisms that potentially contribute to CD development.

## 2. Materials and Methods 

### 2.1. Bacterial Strain

The AIEC reference strain LF82, isolated from a chronic ileal lesion of a CD patient [30], and the non-pathogenic *E. coli* K-12 MG1655 strain were grown in Luria–Bertani (LB) broth overnight at 37 °C without shaking.

### 2.2. Cell Lines and Cell Culture

The human colorectal adenocarcinoma cell line Caco-2 clone 1 ((Caco-2-cl1; ATCC) was cultured in complete Dulbecco’s Modified Eagle Medium (PAA, Pasching, Austria) supplemented with 10% heat-inactivated fetal bovine serum (FBS) (Lonza, Verviers, Belgium), 4 mM L-glutamine (PAA, Pasching, Austria), 100 U/mL penicillin (PAA), and 100 mg/mL streptomycin (PAA, Pasching, Austria). The human Burkitt’s lymphoma cell-line Raji B (ECACC 85011429) was cultured in complete RPMI-1640 medium (PAA, Pasching, Austria), supplemented with 10% heat-inactivated FBS, 8 mM L-glutamine, 100 U/mL penicillin, and 100 mg/mL streptomycin. 

The in vitro M-cell culture model was established as previously described [5]. Briefly, 10^6^ Caco-2-cl1 per mL were seeded onto the apical compartment of Transwell^TM^ filters (Corning, Inc., Corning, NY, USA) previously coated with BD Matrigel^TM^ (Franklin Lakes, NJ, USA). Cells were grown for 17 days until reaching a completely differentiated phenotype. The integrity of cell monolayers was measured by monitoring transepithelial electrical resistance with a Millicell-ERS (Millipore, Burlington, MA, USA). Then, 5 × 10^5^ Raji-B cells were added to the basolateral compartment of the Transwell^TM^ containing the Caco-2-cl1 monolayer, and the co-culture was maintained for 4 to 6 days. Monocultures of Caco-2-cl1 cells on matched filter supports were used as controls. All the cell lines were maintained in a humidified atmosphere at 37 °C containing 5% CO_2_.

### 2.3. Bacterial Translocation across M Cells

For the translocation assay, 10^7^ LF82 bacteria were added into the apical compartment of the M-cell or the control Caco-2-cl1 monolayers. Medium from the basolateral compartment was collected every hour, and 10-fold dilutions were plated onto LB agar plates to determine the number of colony-forming units (CFUs) of translocated bacteria.

### 2.4. Total RNA Isolation from M Cells and Caco-2 Monolayers, Microarray, and miRNA Array

The control Caco-2-cl1 and the M-cell monolayers used for the bacterial translocation assay were collected at 4 h post-infection with the AIEC LF82 strain. The monolayers were washed with cold PBS, and total RNA was extracted using the miRNeasy kit (Hilden, Germany, Qiagen) according to the manufacturer’s protocol. After quantification of RNA concentration by a Nanodrop spectrophotometer (NanoDrop Technologies, Inc., Wilmington, DE, USA) and RNA quality assessment by the Agilent Bioanalyzer (Agilent Biotechnologies, Santa Clara, CA, USA), the same RNA amount was subjected to microarray and miRNA array analyses. Gene expression profiling and data normalization were performed by the ProfileXpert core facility (Lyon, France). Gene expression profiles were analyzed with a whole human genome microarray containing 47,231 probes (HumanHT-12 v4 Expression BeadChip; Illumina Inc., San Diego, CA, USA). Data were normalized by quantile normalization with Genome Studio Software 2010 (Illumina Inc., USA). For miRNA profiling, total RNA was extracted from cells, labeled and hybridized on Affymetrix miRNA GeneChip arrays 3.0, which allow the detection of 1733 human mature miRNA and 1658 human pre-miRNA. After signal extraction, raw data will be normalized to identify miRNA with a significant change in expression.

### 2.5. In Silico Analysis

The prediction of the potential target genes of dysregulated miRNAs was performed using three human miRNA-dedicated algorithms miRanda, miRDB, and Targetscan, analyzed with miRWalk software [31].

## 3. Results

### 3.1. AIEC Bacteria Are Able to Translocate across Human M-Cell Monolayers

**Figure 1:**
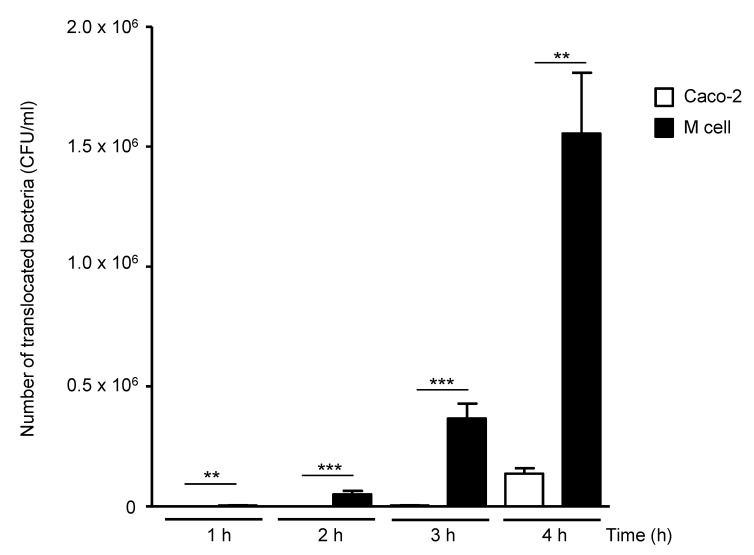
In vitro translocation of AIEC LF82 strain across human M-cell monolayers. A total of 1 × 10^7^ LF82 bacteria were added into the apical compartment of the M-cell or the control Caco-2-cl1 monolayers. The number of translocated bacteria (CFU/mL) into the basolateral compartments of M-cell or Caco-2-cl1 monolayers at 1 to 4 h post-infection was determined. Results are means ± standard error of the mean (SEM) for replicate experiments. ** *p* ≤ 0.01 or *** *p* ≤ 0.001). Statistical analysis was performed using the non-parametric Kruskal–Wallis test with GraphPad Prism version 5.01 software (GraphPad Software, San Diego, CA, USA).

The in vitro M-cell model, obtained by co-culture of human intestinal epithelial Caco-2-cl1 cells with Raji B cells, was used to study AIEC bacterial translocation. Experiments were realized with the AIEC LF82 reference strain. The level of AIEC translocation remained low with the control Caco-2-cl1 monolayers but increased with the M-cell model in a time-dependent manner (Figure 1). The ability of AIEC to translocate across M cells was not due to the reduction of the monolayer integrity since the transepithelial electrical resistance (TEER) remained constant during the time of infection (data not shown).

### 3.2. M-Cell Monolayers Exhibit Distinct miRNA and Gene Profiles Compared to Intestinal Epithelial Caco-2-cl1 Cells

**Figure 2:**
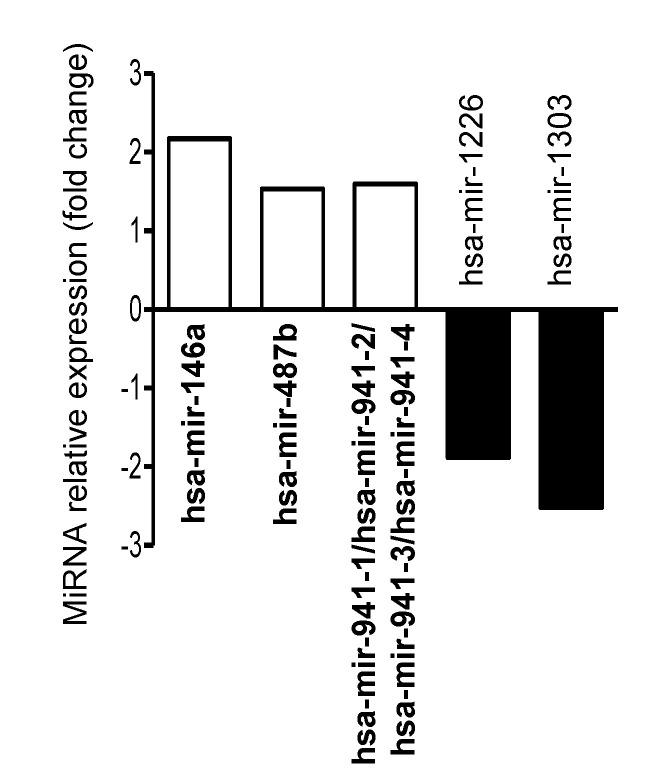
Dysregulated miRNAs in uninfected M cells compared to uninfected Caco-2-cl1 cells. A total of 10^7^ LF82 bacteria were added into the apical compartment of M cells or the Caco-2-cl1 monolayers. At 4 h post-infection, the monolayers were collected, washed with cold PBS, and total RNA was extracted and used for miRNA array analysis. The graph shows the relative expression of the miRNAs that were dysregulated in uninfected M cells versus uninfected Caco-2-cl1 cells, presented as fold change (normalized to expression levels of miRNAs in uninfected Caco-2-cl1 cells, defined as 1).

Since there is a lack of data regarding miRNA-mediated gene regulation in M cells, we performed a comprehensive profiling of miRNAs and genes in M cells in comparison to the control Caco-2-cl1 cells. This revealed that three miRNAs were upregulated and two miRNAs were downregulated in M cells compared to Caco-2-cl1 cells (Figure 2 and Table S1). In silico analysis using the MirWalk (version 3.0) database revealed 1798 potential target genes for three upregulated miRNAs, while 2613 potential target genes were uncovered for two downregulated miRNAs (Table S2). These data were predicted by three different algorithms namely miRDB, miRanda, and TargetScan. These potential target genes were further compared to the genes that were dysregulated (563 upregulated and 497 downregulated genes) in M cells versus Caco-2-cl1 cells analyzed by microarray (Table S3). This showed 60 downregulated and 88 upregulated genes in M cells compared to Caco-2-cl1 cells that were potentially regulated by miRNAs (Table S4).

### 3.3. AIEC Infection Induces Dysregulation of miRNAs in IECs and M Cells

**Figure 3:**
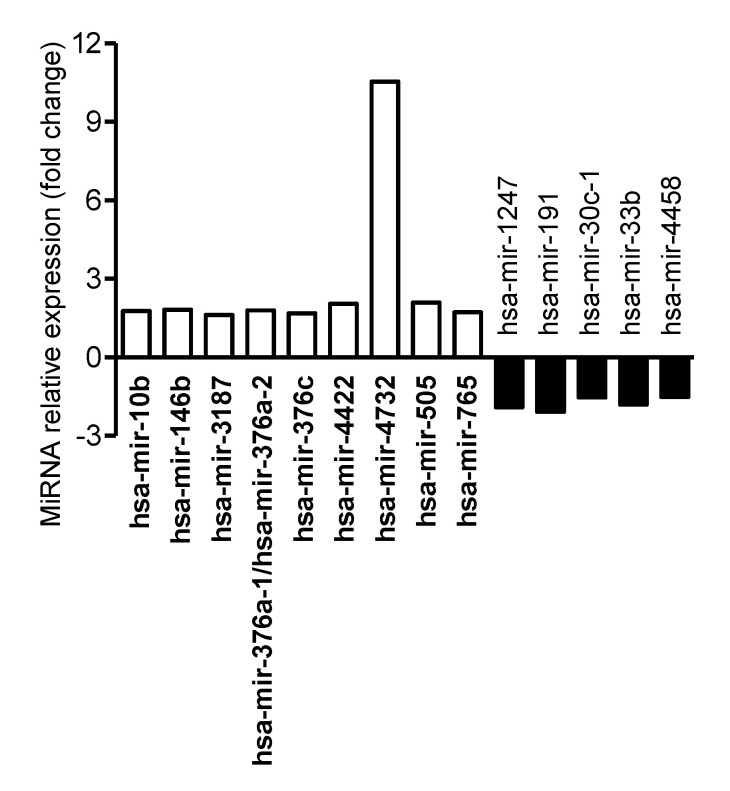
Dysregulated miRNAs in AIEC LF82-infected versus uninfected Caco-2-cl1 cells. A total of 10^7^ LF82 bacteria were added into the apical compartment of Caco-2-cl1 monolayers. At 4 h post-infection, the monolayers were collected, washed with cold PBS, and total RNA was extracted and used for miRNA array analysis. The graph shows the relative expression of the miRNAs that were dysregulated in AIEC LF82-infected versus uninfected Caco-2-cl1 cells, presented as fold change (normalized to expression levels of miRNAs in uninfected Caco-2-cl1 cells, defined as 1).

**Figure 4:**
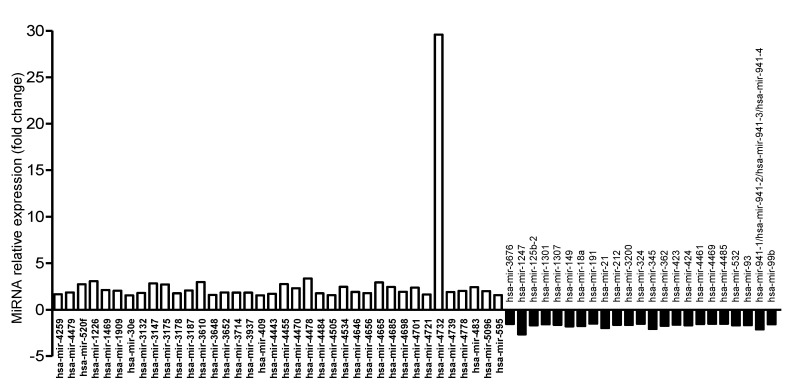
Dysregulated miRNAs in AIEC LF82-infected versus uninfected M cells. A total of 10^7^ LF82 bacteria were added into the apical compartment of M cells. At 4 h post-infection, the monolayers were collected, washed with cold PBS, and total RNA was extracted and used for miRNA array analysis. The graph shows the relative expression of the miRNAs that were dysregulated in AIEC LF82-infected versus uninfected M cells, presented as fold change (normalized to expression levels of miRNAs in uninfected M cells, defined as 1).

Since AIEC bacteria translocate across M cells or, to a lesser extent, IECs to reach the submucosa and invade their host, we investigated how AIEC infection of M cells or Caco-2 monolayers modulates miRNA levels in host cells. M cells or Caco-2 monolayers were infected or not with AIEC LF82 for 4 h. Comparative profiling of miRNA expression revealed that 14 human miRNAs were dysregulated in AIEC LF82-infected versus uninfected Caco-2-cl1 cells (Figure 3 and Table S5). Among these miRNAs, nine were upregulated and five were downregulated (Table S5). In AIEC LF82-infected compared with uninfected M cells, 61 miRNAs were significantly dysregulated, among which 38 were upregulated and 23 were downregulated (Figure 4 and Table S6). A comparison of the dysregulated miRNAs revealed that hsa-miR-1247-5p and hsa-miR-191-3p were downregulated, whereas hsa-miR-3187-3p and hsa-miR-4732-5p were upregulated in both Caco-2-cl1 and M cells in response to AIEC LF82 infection. These results suggested that several miRNAs might contribute to host cell response specifically to AIEC LF82 infection, either in IECs or in M cells.

### 3.4. In Silico Analysis to Predict the Potential Target Genes of Dysregulated miRNAs in IECs and M Cells during AIEC Infection 

The potential target genes of the miRNAs that were dysregulated in response to AIEC LF82 infection in Caco-2-cl1 and M cells were predicted using the MirWalk database. Target genes predicted by three algorithms miRDB, miRanda, and TargetScan were considered. This analysis revealed 4298 potential target genes for the five downregulated miRNAs and 7553 potential target genes for the nine upregulated miRNAs in LF82-infected versus uninfected Caco-2-cl1 cells (Table S7). Concerning M cells, 9605 and 13612 potential target genes were identified for the 23 downregulated and 38 upregulated miRNAs upon AIEC LF82 infection, respectively (Table S8). For the downregulated hsa-miR-4461 and the upregulated hsa-miR-5096, the pattern of RNAseq reads does not support their annotation as miRNAs, therefore, they were removed from miRBase. Hsa-miR-4461 and hsa-miR-5096 were, therefore, removed from our study for potential target genes.

### 3.5. AIEC Infection Induces Gene Dysregulation in IECs and M Cells

To identify the genes that were dysregulated in Caco-2-cl1 and M cells upon AIEC infection, a microarray assay was performed. This revealed that 286 genes were upregulated and 245 genes were downregulated in response to AIEC LF82 infection in Caco-2-cl1 cells (Table S9). In M cells, 1032 genes were upregulated and 886 genes were downregulated upon AIEC LF82 infection (Table S10). Interestingly, 177 upregulated genes and 128 downregulated genes were shared between Caco-2-cl1 and M cells in response to AIEC LF82 infection (Table S11).

### 3.6. MiRNA-Mediated Gene Regulation in IECs and M Cells during AIEC Infection 

Since miRNAs negatively regulate gene expression, to reveal the dysregulated miRNA-gene axis during AIEC infection, the upregulated and downregulated genes identified by microarray were compared with the potential target genes of the downregulated and upregulated miRNAs, respectively. This revealed that in LF82-infected versus uninfected Caco-2-cl1 cells, the downregulated miRNAs potentially target 57 of the 286 upregulated genes, while the upregulated miRNAs target 98 of the 245 downregulated genes (Table S12). In LF82-infected versus uninfected M cells, the downregulated miRNAs potentially target 476 of the 1032 upregulated genes, whereas the upregulated miRNAs potentially target 652 of the 886 downregulated genes (Table S13). These genes, which were dysregulated in Caco-2-cl1 cells or M cells upon LF82 infection and were potentially regulated by miRNAs, were classified into different pathways using the Geneontology PANTHER (Protein ANalysis THrough Evolutionary Relationships) Classification System (http://www.pantherdb.org/) (Figures S1 and S2).

Comparison between the dysregulated genes potentially targeted by the dysregulated miRNAs upon AIEC infection in Caco-2 cells and in M cells revealed that miRNAs potentially target 48 downregulated and 36 upregulated genes in both cell lines (Table S14). These miRNA-gene axes might be specific and may importantly contribute to host responses to AIEC infection.

## 4. Discussion

In CD, initial mucosal lesions commonly occur on FAE in the distal ileum and colonic lymphoid follicles [22,32]. While the FAE effectively forms the interface between the intestinal lymphoid system and the luminal environment, M cells [5,26,29], specialized cells of the FAE overlying PPs, facilitate the uptake of macromolecules, antigens, and microorganisms from the intestinal lumen and their delivery to underlying lymphoid tissues to initiate subsequent mucosal immune responses [33]. Ex vivo studies indicate a defective mucosal barrier to bacteria in the PPs from CD patients. We previously demonstrated that CD-associated AIEC are able to interact with mouse and human PPs and to translocate across M-cell monolayers via LPF [5]. We also reported a higher prevalence of *lpf* operon-harboring AIEC strains in CD patients compared with control subjects [5]. Thus, it is plausible that increased bacterial load across M cells could importantly contribute to the early development of CD. 

While M cells are critical for the entry and dissemination of AIEC bacteria, a comprehensive analysis of gene expression and its key regulators, in particular miRNAs, in M cells in response to AIEC infection remains to be investigated. Here, we performed a comprehensive profiling of genes and miRNAs that were dysregulated in the intestinal epithelial Caco-2-cl1 monolayer and M cells upon infection with the AIEC LF82 strain. These experiments were analyzed via an in silico analysis to identify the genes that are potentially regulated by miRNAs during LF82 infection. 

MiRNA profiling revealed that 14 human miRNAs were dysregulated in AIEC LF82-infected vs. uninfected Caco-2-cl1 cells. Among these miRNAs, an increased level of miR-146b has been previously associated with *Clostridium difficile* [34], *Helicobacter pylori* [35], and *Brucella melitensis* infections [36] and with an inhibition of autophagy activation upon *B. melitensis* infection [36]. An overexpression of miR-146b has also been reported in the inflamed colonic mucosa of CD patients compared to the uninflamed colonic mucosa of both CD patients and control subjects [37]. The level of this miRNA was also increased in the serum of IBD patients compared to healthy subjects [38]. In AIEC LF82-infected compared with uninfected M cells, 38 miRNAs were upregulated and 23 were downregulated. Our approach revealed that 476 upregulated and 652 downregulated genes are potentially regulated by miRNAs in M cells upon LF82 infection. Similarly, in Caco-2-cl1 cells, 57 upregulated and 98 downregulated genes that are potentially regulated by miRNAs upon LF82 infection were identified. Interestingly, following infection with LF82 strain, 36 and 48 potential miRNA-regulated genes were upregulated and downregulated, respectively, in both Caco-2-cl1 and M cells, suggesting that these genes might be specific for host response to AIEC infection. 

Among these genes, we reported an increased expression of *TNFAIP3* (TNF alpha-induced protein 3), coding for the TNFAIP3/A20 E3 ubiquitin ligase. This result is consistent with previous studies reporting an increased expression of *TNFAIP3* in M cells upon *Escherichia *coli** and *Bacteroides fragilis* infection [39] and in both M cells and Caco-2-cl1 cells upon *Salmonella* Typhimurium infection [40]. We hypothesized that the increased expression of *TNFAIP3* may be due to the decreased expression of hsa-miR-4458 in Caco-2-cl1 cells and of hsa-miR-18a-5p and hsa-miR-345-5p in M cells, and may have an impact on the induction of pro-inflammatory responses and the maintenance of intestinal epithelial barrier integrity. Indeed, TNFAIP3 interferes with TLR (Toll-like receptors) and NOD2 (nucleotide-binding oligomerization domain 2) signaling pathways to limit NF-κB activation and pro-inflammatory cytokine production [41,42], which may help infected IECs to limit the pro-inflammatory responses induced by AIEC infection [8,9]. Moreover, several in vivo studies demonstrated that TNFAIP3 promotes the maintenance of the intestinal epithelial barrier in supporting epithelial cell tight junctions [43,44] and in preventing cytokine-induced apoptosis of IECs [45]. Even if a role of TNFAIP3 in the regulation of M cell tight junctions and cytokine-induced apoptosis remains to be investigated, we can assume that the increased expression of *TNFAIP3* gene in Caco-2-cl1 and M cells may participate to the maintenance of the integrity of M cells observed upon AIEC infection. 

Our study also reported an increased expression of the *NAMPT* (nicotinamide phosphoribosyltransferase) gene in response to AIEC infection in both M cells and Caco-2-cl1 cells. In silico analysis revealed that *NAMPT* mRNA is a potential target of hsa-miR-33b-3p, which is decreased in AIEC-infected Caco-2-cl1 cells, and of hsa-miR-3200-3p and hsa-miR-3101-3p, which are decreased in AIEC-infected M cells. An increased level of *NAMPT* mRNA has been reported in organoid cultures of human gastric epithelial cells upon *Helicobacter pylori* infection [46]. In murine and human lung endothelial cells, secreted NAMPT triggers TLR4-mediated activation of the NF-κB signaling pathway and transcription and secretion of pro-inflammatory cytokines [47]. Recently, an overexpression and secretion of NAMPT have been reported in IBD patients at both local and systemic levels and have been associated with inflammation, hypoxia, and tissue repair [48]. Thus, AIEC bacteria may play a role in the increased expression and secretion of NAMPT in IBD and this may be modulated via miRNAs. Further studies are needed to investigate the cellular processes in which these genes are implicated and their function, as well as how they are regulated by miRNAs. This can help to have a full understanding of molecular interactions between AIEC and IECs or AIEC and M cells.

Our study also identified the potentially miRNA-regulated genes that were dysregulated in M cells compared to Caco-2-cl1 cells. Several previous studies have tried to perform gene expression profiling of M cells and IECs under basal condition [49,50]. Among the five miRNAs that were dysregulated in M cells compared to Caco-2-cl1 cells in this study, we found an increased level of hsa-miR-146a. This miRNA has been previously reported to play a role in the maturation of human natural killer cells [51] and Jurkat T-lymphoblasts [52], supporting its implication in M cell differentiation. Furthermore, several genes upregulated in M cells compared to Caco-2-cl1 cells were previously reported to play a role in the differentiation of IECs. Among these genes, *BMP2* (bone morphogenetic protein 2) encodes a protein that promotes the differentiation of mature colonocytes [53] and is associated with increased epithelial cell turnover following intestinal ischemia-reperfusion in rats [54]. Similarly, an increased density of insulin-like growth factor 1 receptor, encoded by *IGF1R* gene, has been shown upon the differentiation of intestinal epithelial Caco-2 cells [55]. Three upregulated genes were previously associated with the differentiation of various cell types, such as *TBX3* (T-box transcription factor 3) and *EOMES* (eomesodermin) driving endodermal differentiation of embryonic stem cells [56] or *MEF2D* (myocyte enhancer factor 2D) involved in neuron [57] and muscle [58] differentiation. Finally, this comprehensive miRNA-gene profiling revealed several genes that have not been previously associated with cell differentiation. Our study emphasizes the existing data on gene expression profiling of M cells and IECs and further provides the potential miRNA regulators of the dysregulated genes. Thus, this could help to have a full understanding of the molecular mechanisms underlying the differentiation of IECs into M cells.

Given the importance of M cells in intestinal physiopathology, especially in pathogenic bacterial infection, this study, although remaining descriptive, gives comprehensive data on gene and miRNA expression in M cells in response to infection with AIEC, which have been increasingly accepted as an etiologic factor of CD. Further functional studies, which are out of the scope of this study, are of importance to reveal the biological pathways, together with their components, that are crucial for host defense to CD-associated AIEC infection.

## 5. Conclusions

This study performs a comprehensive analysis of miRNA and gene profiling in human intestinal epithelial Caco-2 monolayers and in an in vitro M-cell model under basal condition and upon infection with the reference AIEC LF82 strain. Thus, it facilitates the discovery of M cell-specific and AIEC response-specific gene-miRNA signature and enhances the molecular understanding of M cell biology under basal condition and in response to infection with CD-associated AIEC.

## Supplementary Materials

The following are available online at https://www.mdpi.com/2076-2607/8/8/1205/s1, Figure S1: Classification into pathways of the dysregulated genes in LF82-infected vs. uninfected Caco-2-cl1 cells that were potentially regulated by miRNAs, Figure S2: Classification into pathways of the dysregulated genes in LF82-infected versus uninfected M cells that were potentially regulated by miRNAs, Table S1: Dysregulated miRNAs in uninfected M cells compared to uninfected Caco-2-cl1 cells, Table S2: Potential target genes of dysregulated miRNAs in uninfected M cells compared to uninfected Caco-2-cl1 cells, Table S3: Dysregulated genes in uninfected M cells versus uninfected Caco-2-cl1 cells, Table S4: Dysregulated genes in uninfected M cells compared to Caco-2-cl1 cells that were potentially regulated by miRNAs, Table S5: Dysregulated miRNAs in AIEC LF82-infected versus uninfected Caco-2-cl1 cells, Table S6: Dysregulated miRNAs in AIEC LF82-infected versus uninfected M cells, Table S7: Potential target genes of dysregulated miRNAs in LF82-infected compared to uninfected Caco-2-cl1 cells, Table S8: Potential target genes of dysregulated miRNAs in LF82-infected compared to uninfected M cells, Table S9: Dysregulated genes in AIEC LF82-infected versus uninfected Caco-2-cl1 cells, Table S10: Dysregulated genes in AIEC LF82-infected versus uninfected M cells, Table S11: Genes that were dysregulated in both Caco-2-cl1 cells and M cells upon AIEC LF82 infection, Table S12: Dysregulated genes in LF82-infected versus uninfected Caco-2-cl1 cells that were potentially regulated by miRNAs, Table S13: Dysregulated genes in LF82-infected versus uninfected M cells that were potentially regulated by miRNAs, Table S14: Genes that were dysregulated in both Caco-2-cl1 cells and M cells upon AIEC LF82 infection and were potentially targeted by miRNAs.

## Abbreviations

AIEC	Adherent-invasive *E. coli*
ATG16L1	Autophagy related 16 like 1
BMP2	Bone morphogenetic protein 2
Caco-2-cl1	Caco-2 clone 1
CD	Crohn’s disease
CFUs	Colony-forming units
EOMES	Eomesodermin
FAE	Follicle associated epithelium
IECs	Intestinal epithelial cells
IGF	Insulin-like growth factor
LB	Luria–Bertani
LPF	Long polar fimbriae
M cells	Microfold or membranous cells
MEF2D	Myocyte enhancer factor 2d
miRNA, miR	MicroRNA
NAMPT	Nicotinamide phosphoribosyltransferase
NOD2	Nucleotide-binding oligomerization domain 2
SNP	Single nucleotide polymorphism
TBX3	T-box transcription factor 3
TEER	Transepithelial electrical resistance
TLR	Toll-like receptors

## References

[1] Carrière J., Darfeuille-Michaud A., Nguyen H.T.T. (2014). Infectious etiopathogenesis of Crohn’s disease. World J. Gastroenterol..

[2] Øyri S.F., Műzes G., Sipos F. (2015). Dysbiotic gut microbiome: A key element of Crohn’s disease. Comp. Immunol. Microbiol. Infect. Dis..

[3] O’Brien C.L., Bringer M.-A., Holt K.E., Gordon D.M., Dubois A.L., Barnich N., Darfeuille-Michaud A., Pavli P. (2017). Comparative genomics of Crohn’s disease-associated adherent-invasive *Escherichia coli*. Gut.

[4] Darfeuille-Michaud A., Boudeau J., Bulois P., Neut C., Glasser A.-L., Barnich N., Bringer M.-A., Swidsinski A., Beaugerie L., Colombel J.-F. (2004). High prevalence of adherent-invasive *Escherichia coli* associated with ileal mucosa in Crohn’s disease. Gastroenterology.

[5] Chassaing B., Rolhion N., de Vallée A., Salim S.Y., Prorok-Hamon M., Neut C., Campbell B.J., Söderholm J.D., Hugot J., Colombel J.-F. (2011). Crohn disease–associated adherent-invasive *E. coli* bacteria target mouse and human Peyer’s patches via long polar fimbriae. J. Clin. Investig..

[6] Glasser A.L., Boudeau J., Barnich N., Perruchot M.H., Colombel J.F., Darfeuille-Michaud A. (2001). Adherent invasive *Escherichia coli* strains from patients with Crohn’s disease survive and replicate within macrophages without inducing host cell death. Infect. Immun..

[7] Vazeille E., Buisson A., Bringer M.A., Goutte M., Ouchchane L., Hugot J.P., de Vallée A., Barnich N., Bommelaer G., Darfeuille-Michaud A. (2015). Monocyte-derived macrophages from Crohn’s disease patients are impaired in the ability to control intracellular adherent-invasive *Escherichia coli* and exhibit disordered cytokine secretion profile. J. Crohn Colitis.

[8] Nguyen H.T.T., Dalmasso G., Müller S., Carrière J., Seibold F., Darfeuille-Michaud A. (2014). Crohn’s disease-associated adherent invasive escherichia coli modulate levels of microRNAs in intestinal epithelial cells to reduce autophagy. Gastroenterology.

[9] Lapaquette P., Glasser A.-L., Huett A., Xavier R.J., Darfeuille-Michaud A. (2010). Crohn’s disease-associated adherent-invasive *E. coli* are selectively favoured by impaired autophagy to replicate intracellularly. Cell. Microbiol..

[10] Lapaquette P., Bringer M.-A., Darfeuille-Michaud A. (2012). Defects in autophagy favour adherent-invasive *Escherichia coli* persistence within macrophages leading to increased pro-inflammatory response. Cell. Microbiol..

[11] Carvalho F.A., Barnich N., Sivignon A., Darcha C., Chan C.H.F., Stanners C.P., Darfeuille-Michaud A. (2009). Crohn’s disease adherent-invasive *Escherichia coli* colonize and induce strong gut inflammation in transgenic mice expressing human CEACAM. J. Exp. Med..

[12] Martinez-Medina M., Denizot J., Dreux N., Robin F., Billard E., Bonnet R., Darfeuille-Michaud A., Barnich N. (2014). Western diet induces dysbiosis with increased e coli in CEABAC10 mice, alters host barrier function favouring AIEC colonisation. Gut.

[13] Bretin A., Lucas C., Larabi A., Dalmasso G., Billard E., Barnich N., Bonnet R., Nguyen H.T.T. (2018). AIEC infection triggers modification of gut microbiota composition in genetically predisposed mice, contributing to intestinal inflammation. Sci. Rep..

[14] Bretin A., Carrière J., Dalmasso G., Bergougnoux A., B’chir W., Maurin A.C., Müller S., Seibold F., Barnich N., Bruhat A. (2016). Activation of the EIF2AK4-EIF2A/eIF2α-ATF4 pathway triggers autophagy response to Crohn disease-associated adherent-invasive *Escherichia coli* infection. Autophagy.

[15] Dalmasso G., Nguyen H.T.T., Faïs T., Massier S., Barnich N., Delmas J., Bonnet R. (2019). Crohn’s Disease-Associated Adherent-Invasive *Escherichia coli* Manipulate Host Autophagy by Impairing SUMOylation. Cells.

[16] Larabi A., Barnich N., Nguyen H.T.T. (2019). New insights into the interplay between autophagy, gut microbiota and inflammatory responses in IBD. Autophagy.

[17] Carrière J., Bretin A., Darfeuille-Michaud A., Barnich N., Nguyen H.T.T. (2016). Exosomes Released from Cells Infected with Crohnʼs Disease–associated Adherent-Invasive *Escherichia coli* Activate Host Innate Immune Responses and Enhance Bacterial Intracellular Replication. Inflamm. Bowel Dis..

[18] Larabi A., Dalmasso G., Delmas J., Barnich N., Nguyen H.T.T. (2020). Exosomes transfer miRNAs from cell-to-cell to inhibit autophagy during infection with Crohn’s disease-associated adherent-invasive *E. coli*. Gut Microbes.

[19] Moein S., Vaghari-Tabari M., Qujeq D., Majidinia M., Nabavi S.M., Yousefi B. (2019). MiRNAs and inflammatory bowel disease: An interesting new story. J. Cell. Physiol..

[20] Brest P., Lapaquette P., Souidi M., Lebrigand K., Cesaro A., Vouret-Craviari V., Mari B., Barbry P., Mosnier J.-F., Hébuterne X. (2011). A synonymous variant in IRGM alters a binding site for miR-196 and causes deregulation of IRGM-dependent xenophagy in Crohn’s disease. Nat. Genet..

[21] Morson B.C. (1972). The early histological lesion of Crohn’s disease. Proc. R. Soc. Med..

[22] Gullberg E., Söderholm J.D. (2006). Peyer’s patches and M cells as potential sites of the inflammatory onset in Crohn’s disease. Ann. N. Y. Acad. Sci..

[23] Nakamura Y., Kimura S., Hase K. (2018). M cell-dependent antigen uptake on follicle-associated epithelium for mucosal immune surveillance. Inflamm. Regen..

[24] Keita Å., Salim S., Jiang T., Yang P.-C., Franzén L., Söderkvist P., Magnusson K.-E., Söderholm J. (2008). Increased uptake of non-pathogenic *E. coli* via the follicle-associated epithelium in longstanding ileal Crohn’s disease. J. Pathol..

[25] Salim S.Y., Silva M.A., Keita Å.V., Larsson M., Andersson P., Magnusson K.-E., Perdue M.H., Söderholm J.D. (2009). CD83+CCR7− Dendritic Cells Accumulate in the Subepithelial Dome and Internalize Translocated *Escherichia coli* HB101 in the Peyer’s Patches of Ileal Crohn’s Disease. Am. J. Pathol..

[26] Vazeille E., Chassaing B., Buisson A., Dubois A., De Vallée A., Billard E., Neut C., Bommelaer G., Colombel J.F., Barnich N. (2016). GipA Factor Supports Colonization of Peyer’s Patches by Crohn’s Disease-associated *Escherichia coli*. Inflamm. Bowel Dis..

[27] Roberts C.L., Keita A.V., Duncan S.H., O’Kennedy N., Soderholm J.D., Rhodes J.M., Campbell B.J. (2010). Translocation of Crohn’s disease *Escherichia coli* across M-cells: Contrasting effects of soluble plant fibres and emulsifiers. Gut.

[28] Etienne-Mesmin L., Chassaing B., Sauvanet P., Denizot J., Blanquet-Diot S., Darfeuille-Michaud A., Pradel N., Livrelli V. (2011). Interactions with M cells and macrophages as key steps in the pathogenesis of enterohemorragic *Escherichia coli* infections. PLoS ONE.

[29] Dogan B., Suzuki H., Herlekar D., Sartor B.R.B., Campbell B.J., Roberts C.L., Stewart K., Scherl E.J., Araz Y., Bitar P.P. (2014). Inflammation-associated adherent-invasive escherichia coli are enriched in pathways for use of propanediol and iron and M-cell translocation. Inflamm. Bowel Dis..

[30] Darfeuille-Michaud A., Neut C., Barnich N., Lederman E., Di Martino P., Desreumaux P., Gambiez L., Joly B., Cortot A., Colombel J.-F. (1998). Presence of adherent *Escherichia coli* strains in ileal mucosa of patients with Crohn’s disease. Gastroenterology.

[31] Dweep H., Sticht C., Pandey P., Gretz N. (2011). miRWalk—Database: Prediction of possible miRNA binding sites by “walking” the genes of three genomes. J. Biomed. Inform..

[32] Fujimura Y., Kamoi R., Iida M. (1996). Pathogenesis of aphthoid ulcers in Crohn’s disease: Correlative findings by magnifying colonoscopy, electron microscopy, and immunohistochemistry. Gut.

[33] Da Silva C., Wagner C., Bonnardel J., Gorvel J.P., Lelouard H. (2017). The Peyer’s patch mononuclear phagocyte system at steady state and during infection. Front. Immunol..

[34] Viladomiu M., Hontecillas R., Pedragosa M., Carbo A., Hoops S., Michalak P., Michalak K., Guerrant R.L., Roche J.K., Warren C.A. (2012). Modeling the Role of Peroxisome Proliferator-Activated Receptor γ and MicroRNA-146 in Mucosal Immune Responses to Clostridium difficile. PLoS ONE.

[35] Cheng S.F., Li L., Wang L.M. (2015). miR-155 and miR-146b negatively regulates IL6 in Helicobacter pylori (cagA+) infected gastroduodenal ulcer. Eur. Rev. Med. Pharmacol. Sci..

[36] Hanwei J., Nie X., Zhu H., Li B., Pang F., Yang X., Cao R., Yang X., Zhu S., Peng D. (2020). miR-146b-5p Plays a Critical Role in the Regulation of Autophagy in ∆per Brucella melitensis -Infected RAW264.7 Cells. Biomed Res. Int..

[37] Wu L.Y., Ma X.P., Shi Y., Bao C.H., Jin X.M., Lu Y., Zhao J.M., Zhou C.L., Chen D., Liu H.R. (2017). Alterations in microRNA expression profiles in inflamed and noninflamed ascending colon mucosae of patients with active Crohn’s disease. J. Gastroenterol. Hepatol..

[38] Chen P., Li Y., Li L., Yu Q., Chao K., Zhou G., Qiu Y., Feng R., Huang S., He Y. (2019). Circulating microRNA146b-5p is superior to C-reactive protein as a novel biomarker for monitoring inflammatory bowel disease. Aliment. Pharmacol. Ther..

[39] Lapthorne S., MacSharry J., Scully P., Nally K., Shanahan F. (2012). Differential intestinal M-cell gene expression response to gut commensals. Immunology.

[40] Wang K.-C., Huang C.-H., Huang C.-J., Fang S.-B. (2016). Impacts of Salmonella enterica Serovar Typhimurium and Its speG Gene on the Transcriptomes of In Vitro M Cells and Caco-2 Cells. PLoS ONE.

[41] Hitotsumatsu O., Ahmad R.-C., Tavares R., Wang M., Philpott D., Turer E.E., Lee B.L., Shiffin N., Advincula R., Malynn B.A. (2008). The Ubiquitin-Editing Enzyme A20 Restricts Nucleotide-Binding Oligomerization Domain Containing 2-Triggered Signals. Immunity.

[42] Oshima N., Ishihara S., Rumi M.A.K., Aziz M.M., Mishima Y., Kadota C., Moriyama I., Ishimura N., Amano Y., Kinoshita Y. (2010). A20 is an early responding negative regulator of Toll-like receptor 5 signalling in intestinal epithelial cells during inflammation. Clin. Exp. Immunol..

[43] Kolodziej L.E., Lodolce J.P., Chang J.E., Schneider J.R., Grimm W.A., Bartulis S.J., Zhu X., Messer J.S., Murphy S.F., Reddy N. (2011). TNFAIP3 Maintains Intestinal Barrier Function and Supports Epithelial Cell Tight Junctions. PLoS ONE.

[44] Shi Y., Guo Y., Zhou J., Wu L., Chen L., Sun Y., Li T., Zhao J., Bao C., Wu H. (2019). Herbs-partitioned moxibustion improves intestinal epithelial tight junctions by upregulating A20 expression in a mouse model of Crohn’s disease. Biomed. Pharmacother..

[45] Vereecke L., Vieira-Silva S., Billiet T., van Es J.H., Mc Guire C., Slowicka K., Sze M., van den Born M., De Hertogh G., Clevers H. (2014). A20 controls intestinal homeostasis through cell-specific activities. Nat. Commun..

[46] Bartfeld S., Bayram T., van de Wetering M., Huch M., Begthel H., Kujala P., Vries R., Peters P.J., Clevers H. (2015). In Vitro Expansion of Human Gastric Epithelial Stem Cells and Their Responses to Bacterial Infection. Gastroenterology.

[47] Camp S.M., Ceco E., Evenoski C.L., Danilov S.M., Zhou T., Chiang E.T., Moreno-Vinasco L., Mapes B., Zhao J., Gursoy G. (2015). Unique Toll-Like Receptor 4 Activation by NAMPT/PBEF Induces NFκ B Signaling and Inflammatory Lung Injury. Sci. Rep..

[48] Neubauer K., Bednarz-Misa I., Walecka-Zacharska E., Wierzbicki J., Agrawal A., Gamian A., Krzystek-Korpacka M. (2019). Oversecretion and Overexpression of Nicotinamide Phosphoribosyltransferase/Pre-B Colony-Enhancing Factor/Visfatin in Inflammatory Bowel Disease Reflects the Disease Activity, Severity of Inflammatory Response and Hypoxia. Int. J. Mol. Sci..

[49] Terahara K., Yoshida M., Igarashi O., Nochi T., Pontes G.S., Hase K., Ohno H., Kurokawa S., Mejima M., Takayama N. (2008). Comprehensive gene expression profiling of Peyer’s patch M cells, villous M-like cells, and intestinal epithelial cells. J. Immunol..

[50] Nakato G., Fukuda S., Hase K., Goitsuka R., Cooper M.D., Ohno H. (2009). New Approach for M-Cell-Specific Molecules Screening by Comprehensive Transcriptome Analysis. DNA Res..

[51] Pesce S., Squillario M., Greppi M., Loiacono F., Moretta L., Moretta A., Sivori S., Castagnola P., Barla A., Candiani S. (2018). New miRNA Signature Heralds Human NK Cell Subsets at Different Maturation Steps: Involvement of miR-146a-5p in the Regulation of KIR Expression. Front. Immunol..

[52] Saki N., Abroun S., Soleimani M., Mortazavi Y., Kaviani S., Arefian E. (2014). The roles of miR-146a in the differentiation of Jurkat T-lymphoblasts. Hematology.

[53] Hardwick J.C.H., Van Den Brink G.R., Bleuming S.A., Ballester I., Van Den Brande J.M.H., Keller J.J., Offerhaus G.J.A., Van Deventer S.J.H., Peppelenbosch M.P. (2004). Bone morphogenetic protein 2 is expressed by, and acts upon, mature epithelial cells in the colon. Gastroenterology.

[54] Ben-Shahar Y., Abassi Z., Kreizman Shefer H., Pollak Y., Bhattacharya U., Sukhotnik I. (2020). Accelerated Intestinal Epithelial Cell Turnover Correlates with Stimulated BMP Signaling Cascade following Intestinal Ischemia–Reperfusion in a Rat. Eur. J. Pediatr. Surg..

[55] Oguchi S., Walker W.A., Sanderson I.R. (1995). Differentiation and Polarity Alter the Binding of IGF-I to Human Intestinal Epithelial (Caco-2) Cells. J. Pediatr. Gastroenterol. Nutr..

[56] Kartikasari A.E.R., Zhou J.X., Kanji M.S., Chan D.N., Sinha A., Grapin-Botton A., Magnuson M.A., Lowry W.E., Bhushan A. (2013). The histone demethylase Jmjd3 sequentially associates with the transcription factors Tbx3 and Eomes to drive endoderm differentiation. EMBO J..

[57] Lam B.Y.H., Chawla S. (2007). MEF2D expression increases during neuronal differentiation of neural progenitor cells and correlates with neurite length. Neurosci. Lett..

[58] Runfola V., Sebastian S., Dilworth F.J., Gabellini D. (2015). Rbfox proteins regulate tissue-specific alternative splicing of Mef2D required for muscle differentiation. J. Cell Sci..

