# Supplementary material for: Differential miRNA-Gene Expression in M Cells in Response to Crohn’s Disease-Associated AIEC
Source: Microorganisms. 2020 Aug 7;8(8):1205. doi: 10.3390/microorganisms8081205 (PMC7466023; doi:10.3390/microorganisms8081205)
Supplement: Supplementary file 1 [file microorganisms-08-01205-s001.zip › Supplementary figures.docx]

*Supplementary material*

Differential miRNA-gene expression in M cells in response to Crohn disease-associated AIEC

Anaïs Larabi ^1^, Laurène Salesse ^1^, Charlotte Cordonnier ^1^, Lucie Etienne-Mesmin ^2^, Nicolas Barnich ^1^, Guillaume Dalmasso ^1^, Hang Thi Thu Nguyen ^1,^*

^1^ M2iSH, UMR 1071 Inserm, Université Clermont Auvergne, INRAE USC 2018, CRNH, Clermont-Ferrand 63001, France; [anais.larabi@gmail.com](mailto:anais.larabi@gmail.com) (A.L), [laurene.salesse@uca.fr](mailto:laurene.salesse@uca.fr) (L.S), [cordonnier.charlotte@wanadoo.fr](mailto:cordonnier.charlotte@wanadoo.fr) (C.C), [nicolas.barnich@uca.fr](mailto:nicolas.barnich@uca.fr) (N.B), [guillaume.dalmasso@uca.fr](mailto:guillaume.dalmasso@uca.fr) (G.D), [hang.nguyen@uca.fr](mailto:hang.nguyen@uca.fr) (H.N)

^2^ MEDIS, INRAE, Université Clermont Auvergne, Clermont-Ferrand 63001, France; lucie.etienne-mesmin@uca.fr

***** Correspondence: Hang Thi Thu Nguyen, PhD. M2iSH, UMR 1071 Inserm, Université Clermont Auvergne, INRA USC 2018, Clermont-Ferrand 63001, France. Tel: (+33) 4 73 17 83 72. Fax: (+33) 4 73 17 83 71. E-mail: [hang.nguyen@uca.fr](mailto:hang.nguyen@uca.fr).


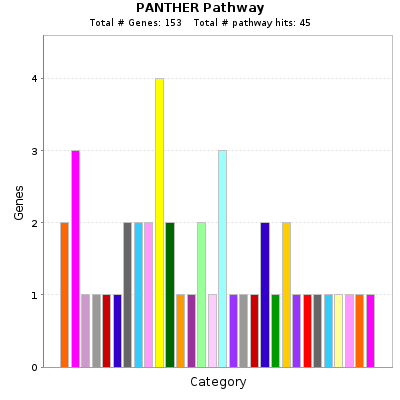


| \| [](javascript:popup('colorPallete.jsp?catSelection=P00005');) \| \| --- \| | [Angiogenesis (P00005)](http://www.pantherdb.org/list/list.do?chartCategoryAcc=P00005&chartCategoryType=3&filterLevel=1&listType=1) |
| --- | --- | --- |
| \| [](javascript:popup('colorPallete.jsp?catSelection=P00006');) \| \| --- \| | [Apoptosis signaling pathway (P00006)](http://www.pantherdb.org/list/list.do?chartCategoryAcc=P00006&chartCategoryType=3&filterLevel=1&listType=1) |
| \| [](javascript:popup('colorPallete.jsp?catSelection=P00008');) \| \| --- \| | [Axon guidance mediated by Slit/Robo (P00008)](http://www.pantherdb.org/list/list.do?chartCategoryAcc=P00008&chartCategoryType=3&filterLevel=1&listType=1) |
| \| [](javascript:popup('colorPallete.jsp?catSelection=P00009');) \| \| --- \| | [Axon guidance mediated by netrin (P00009)](http://www.pantherdb.org/list/list.do?chartCategoryAcc=P00009&chartCategoryType=3&filterLevel=1&listType=1) |
| \| [](javascript:popup('colorPallete.jsp?catSelection=P00010');) \| \| --- \| | [B cell activation (P00010)](http://www.pantherdb.org/list/list.do?chartCategoryAcc=P00010&chartCategoryType=3&filterLevel=1&listType=1) |
| \| [](javascript:popup('colorPallete.jsp?catSelection=P06959');) \| \| --- \| | [CCKR signaling map (P06959)](http://www.pantherdb.org/list/list.do?chartCategoryAcc=P06959&chartCategoryType=3&filterLevel=1&listType=1) |
| \| [](javascript:popup('colorPallete.jsp?catSelection=P00016');) \| \| --- \| | [Cytoskeletal regulation by Rho GTPase (P00016)](http://www.pantherdb.org/list/list.do?chartCategoryAcc=P00016&chartCategoryType=3&filterLevel=1&listType=1) |
| \| [](javascript:popup('colorPallete.jsp?catSelection=P00018');) \| \| --- \| | [EGF receptor signaling pathway (P00018)](http://www.pantherdb.org/list/list.do?chartCategoryAcc=P00018&chartCategoryType=3&filterLevel=1&listType=1) |
| \| [](javascript:popup('colorPallete.jsp?catSelection=P00021');) \| \| --- \| | [FGF signaling pathway (P00021)](http://www.pantherdb.org/list/list.do?chartCategoryAcc=P00021&chartCategoryType=3&filterLevel=1&listType=1) |
| \| [](javascript:popup('colorPallete.jsp?catSelection=P06664');) \| \| --- \| | [Gonadotropin-releasing hormone receptor pathway (P06664)](http://www.pantherdb.org/list/list.do?chartCategoryAcc=P06664&chartCategoryType=3&filterLevel=1&listType=1) |
| \| [](javascript:popup('colorPallete.jsp?catSelection=P00025');) \| \| --- \| | [Hedgehog signaling pathway (P00025)](http://www.pantherdb.org/list/list.do?chartCategoryAcc=P00025&chartCategoryType=3&filterLevel=1&listType=1) |
| \| [](javascript:popup('colorPallete.jsp?catSelection=P00026');) \| \| --- \| | [Heterotrimeric G-protein signaling pathway-Gi alpha and Gs alpha mediated pathway (P00026)](http://www.pantherdb.org/list/list.do?chartCategoryAcc=P00026&chartCategoryType=3&filterLevel=1&listType=1) |
| \| [](javascript:popup('colorPallete.jsp?catSelection=P00027');) \| \| --- \| | [Heterotrimeric G-protein signaling pathway-Gq alpha and Go alpha mediated pathway (P00027)](http://www.pantherdb.org/list/list.do?chartCategoryAcc=P00027&chartCategoryType=3&filterLevel=1&listType=1) |
| \| [](javascript:popup('colorPallete.jsp?catSelection=P00031');) \| \| --- \| | [Inflammation mediated by chemokine and cytokine signaling pathway (P00031)](http://www.pantherdb.org/list/list.do?chartCategoryAcc=P00031&chartCategoryType=3&filterLevel=1&listType=1) |
| \| [](javascript:popup('colorPallete.jsp?catSelection=P00033');) \| \| --- \| | [Insulin/IGF pathway-protein kinase B signaling cascade (P00033)](http://www.pantherdb.org/list/list.do?chartCategoryAcc=P00033&chartCategoryType=3&filterLevel=1&listType=1) |
| \| [](javascript:popup('colorPallete.jsp?catSelection=P00034');) \| \| --- \| | [Integrin signalling pathway (P00034)](http://www.pantherdb.org/list/list.do?chartCategoryAcc=P00034&chartCategoryType=3&filterLevel=1&listType=1) |
| \| [](javascript:popup('colorPallete.jsp?catSelection=P00036');) \| \| --- \| | [Interleukin signaling pathway (P00036)](http://www.pantherdb.org/list/list.do?chartCategoryAcc=P00036&chartCategoryType=3&filterLevel=1&listType=1) |
| \| [](javascript:popup('colorPallete.jsp?catSelection=P00045');) \| \| --- \| | [Notch signaling pathway (P00045)](http://www.pantherdb.org/list/list.do?chartCategoryAcc=P00045&chartCategoryType=3&filterLevel=1&listType=1) |
| \| [](javascript:popup('colorPallete.jsp?catSelection=P00046');) \| \| --- \| | [Oxidative stress response (P00046)](http://www.pantherdb.org/list/list.do?chartCategoryAcc=P00046&chartCategoryType=3&filterLevel=1&listType=1) |
| \| [](javascript:popup('colorPallete.jsp?catSelection=P00047');) \| \| --- \| | [PDGF signaling pathway (P00047)](http://www.pantherdb.org/list/list.do?chartCategoryAcc=P00047&chartCategoryType=3&filterLevel=1&listType=1) |
| \| [](javascript:popup('colorPallete.jsp?catSelection=P00048');) \| \| --- \| | [PI3 kinase pathway (P00048)](http://www.pantherdb.org/list/list.do?chartCategoryAcc=P00048&chartCategoryType=3&filterLevel=1&listType=1) |
| \| [](javascript:popup('colorPallete.jsp?catSelection=P00049');) \| \| --- \| | [Parkinson disease (P00049)](http://www.pantherdb.org/list/list.do?chartCategoryAcc=P00049&chartCategoryType=3&filterLevel=1&listType=1) |
| \| [](javascript:popup('colorPallete.jsp?catSelection=P02772');) \| \| --- \| | [Pyruvate metabolism (P02772)](http://www.pantherdb.org/list/list.do?chartCategoryAcc=P02772&chartCategoryType=3&filterLevel=1&listType=1) |
| \| [](javascript:popup('colorPallete.jsp?catSelection=P04393');) \| \| --- \| | [Ras Pathway (P04393)](http://www.pantherdb.org/list/list.do?chartCategoryAcc=P04393&chartCategoryType=3&filterLevel=1&listType=1) |
| \| [](javascript:popup('colorPallete.jsp?catSelection=P00052');) \| \| --- \| | [TGF-beta signaling pathway (P00052)](http://www.pantherdb.org/list/list.do?chartCategoryAcc=P00052&chartCategoryType=3&filterLevel=1&listType=1) |
| \| [](javascript:popup('colorPallete.jsp?catSelection=P00054');) \| \| --- \| | [Toll receptor signaling pathway (P00054)](http://www.pantherdb.org/list/list.do?chartCategoryAcc=P00054&chartCategoryType=3&filterLevel=1&listType=1) |
| \| [](javascript:popup('colorPallete.jsp?catSelection=P00056');) \| \| --- \| | [VEGF signaling pathway (P00056)](http://www.pantherdb.org/list/list.do?chartCategoryAcc=P00056&chartCategoryType=3&filterLevel=1&listType=1) |
| \| [](javascript:popup('colorPallete.jsp?catSelection=P00057');) \| \| --- \| | [Wnt signaling pathway (P00057)](http://www.pantherdb.org/list/list.do?chartCategoryAcc=P00057&chartCategoryType=3&filterLevel=1&listType=1) |
| \| [](javascript:popup('colorPallete.jsp?catSelection=P04397');) \| \| --- \| | [p53 pathway by glucose deprivation (P04397)](http://www.pantherdb.org/list/list.do?chartCategoryAcc=P04397&chartCategoryType=3&filterLevel=1&listType=1) |
| \| [](javascript:popup('colorPallete.jsp?catSelection=P04398');) \| \| --- \| | [p53 pathway feedback loops 2 (P04398)](http://www.pantherdb.org/list/list.do?chartCategoryAcc=P04398&chartCategoryType=3&filterLevel=1&listType=1) |

**Figure S1.** **Classification into pathways of the dysregulated genes in LF82-infected *versus* uninfected Caco-2-cl1 cells that were potentially regulated by miRNAs.** The GENEONTOLOGY PANTHER (Protein ANalysis THrough Evolutionary Relationships) Classification System was used:

(<http://www.pantherdb.org/chart/pantherChart.jsp?listType=1&filterLevel=1&type=3&chartType=2&save=yes&basketItems=all&zoom=1&trackingId=042BE96F5118881E0C45A6320456E8B4>).


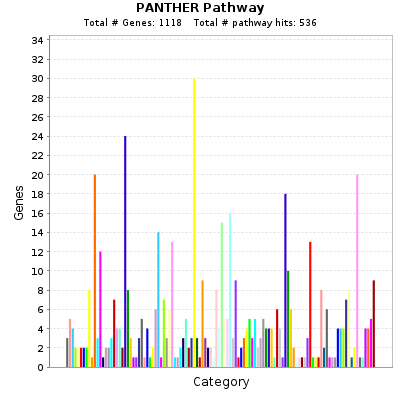


| \| [](javascript:popup('colorPallete.jsp?catSelection=P04372');) \| \| --- \| | [5-Hydroxytryptamine degredation (P04372)](http://www.pantherdb.org/list/list.do?chartCategoryAcc=P04372&chartCategoryType=3&filterLevel=1&listType=1) |
| --- | --- | --- |
| \| [](javascript:popup('colorPallete.jsp?catSelection=P04373');) \| \| --- \| | [5HT1 type receptor mediated signaling pathway (P04373)](http://www.pantherdb.org/list/list.do?chartCategoryAcc=P04373&chartCategoryType=3&filterLevel=1&listType=1) |
| \| [](javascript:popup('colorPallete.jsp?catSelection=P04374');) \| \| --- \| | [5HT2 type receptor mediated signaling pathway (P04374)](http://www.pantherdb.org/list/list.do?chartCategoryAcc=P04374&chartCategoryType=3&filterLevel=1&listType=1) |
| \| [](javascript:popup('colorPallete.jsp?catSelection=P04375');) \| \| --- \| | [5HT3 type receptor mediated signaling pathway (P04375)](http://www.pantherdb.org/list/list.do?chartCategoryAcc=P04375&chartCategoryType=3&filterLevel=1&listType=1) |
| \| [](javascript:popup('colorPallete.jsp?catSelection=P04376');) \| \| --- \| | [5HT4 type receptor mediated signaling pathway (P04376)](http://www.pantherdb.org/list/list.do?chartCategoryAcc=P04376&chartCategoryType=3&filterLevel=1&listType=1) |
| \| [](javascript:popup('colorPallete.jsp?catSelection=P00001');) \| \| --- \| | [Adrenaline and noradrenaline biosynthesis (P00001)](http://www.pantherdb.org/list/list.do?chartCategoryAcc=P00001&chartCategoryType=3&filterLevel=1&listType=1) |
| \| [](javascript:popup('colorPallete.jsp?catSelection=P00002');) \| \| --- \| | [Alpha adrenergic receptor signaling pathway (P00002)](http://www.pantherdb.org/list/list.do?chartCategoryAcc=P00002&chartCategoryType=3&filterLevel=1&listType=1) |
| \| [](javascript:popup('colorPallete.jsp?catSelection=P00003');) \| \| --- \| | [Alzheimer disease-amyloid secretase pathway (P00003)](http://www.pantherdb.org/list/list.do?chartCategoryAcc=P00003&chartCategoryType=3&filterLevel=1&listType=1) |
| \| [](javascript:popup('colorPallete.jsp?catSelection=P00004');) \| \| --- \| | [Alzheimer disease-presenilin pathway (P00004)](http://www.pantherdb.org/list/list.do?chartCategoryAcc=P00004&chartCategoryType=3&filterLevel=1&listType=1) |
| \| [](javascript:popup('colorPallete.jsp?catSelection=P02726');) \| \| --- \| | [Aminobutyrate degradation (P02726)](http://www.pantherdb.org/list/list.do?chartCategoryAcc=P02726&chartCategoryType=3&filterLevel=1&listType=1) |
| \| [](javascript:popup('colorPallete.jsp?catSelection=P00005');) \| \| --- \| | [Angiogenesis (P00005)](http://www.pantherdb.org/list/list.do?chartCategoryAcc=P00005&chartCategoryType=3&filterLevel=1&listType=1) |
| \| [](javascript:popup('colorPallete.jsp?catSelection=P05911');) \| \| --- \| | [Angiotensin II-stimulated signaling through G proteins and beta-arrestin (P05911)](http://www.pantherdb.org/list/list.do?chartCategoryAcc=P05911&chartCategoryType=3&filterLevel=1&listType=1) |
| \| [](javascript:popup('colorPallete.jsp?catSelection=P00006');) \| \| --- \| | [Apoptosis signaling pathway (P00006)](http://www.pantherdb.org/list/list.do?chartCategoryAcc=P00006&chartCategoryType=3&filterLevel=1&listType=1) |
| \| [](javascript:popup('colorPallete.jsp?catSelection=P02728');) \| \| --- \| | [Arginine biosynthesis (P02728)](http://www.pantherdb.org/list/list.do?chartCategoryAcc=P02728&chartCategoryType=3&filterLevel=1&listType=1) |
| \| [](javascript:popup('colorPallete.jsp?catSelection=P00008');) \| \| --- \| | [Axon guidance mediated by Slit/Robo (P00008)](http://www.pantherdb.org/list/list.do?chartCategoryAcc=P00008&chartCategoryType=3&filterLevel=1&listType=1) |
| \| [](javascript:popup('colorPallete.jsp?catSelection=P00009');) \| \| --- \| | [Axon guidance mediated by netrin (P00009)](http://www.pantherdb.org/list/list.do?chartCategoryAcc=P00009&chartCategoryType=3&filterLevel=1&listType=1) |
| \| [](javascript:popup('colorPallete.jsp?catSelection=P00007');) \| \| --- \| | [Axon guidance mediated by semaphorins (P00007)](http://www.pantherdb.org/list/list.do?chartCategoryAcc=P00007&chartCategoryType=3&filterLevel=1&listType=1) |
| \| [](javascript:popup('colorPallete.jsp?catSelection=P00010');) \| \| --- \| | [B cell activation (P00010)](http://www.pantherdb.org/list/list.do?chartCategoryAcc=P00010&chartCategoryType=3&filterLevel=1&listType=1) |
| \| [](javascript:popup('colorPallete.jsp?catSelection=P04377');) \| \| --- \| | [Beta1 adrenergic receptor signaling pathway (P04377)](http://www.pantherdb.org/list/list.do?chartCategoryAcc=P04377&chartCategoryType=3&filterLevel=1&listType=1) |
| \| [](javascript:popup('colorPallete.jsp?catSelection=P04378');) \| \| --- \| | [Beta2 adrenergic receptor signaling pathway (P04378)](http://www.pantherdb.org/list/list.do?chartCategoryAcc=P04378&chartCategoryType=3&filterLevel=1&listType=1) |
| \| [](javascript:popup('colorPallete.jsp?catSelection=P04379');) \| \| --- \| | [Beta3 adrenergic receptor signaling pathway (P04379)](http://www.pantherdb.org/list/list.do?chartCategoryAcc=P04379&chartCategoryType=3&filterLevel=1&listType=1) |
| \| [](javascript:popup('colorPallete.jsp?catSelection=P06959');) \| \| --- \| | [CCKR signaling map (P06959)](http://www.pantherdb.org/list/list.do?chartCategoryAcc=P06959&chartCategoryType=3&filterLevel=1&listType=1) |
| \| [](javascript:popup('colorPallete.jsp?catSelection=P00012');) \| \| --- \| | [Cadherin signaling pathway (P00012)](http://www.pantherdb.org/list/list.do?chartCategoryAcc=P00012&chartCategoryType=3&filterLevel=1&listType=1) |
| \| [](javascript:popup('colorPallete.jsp?catSelection=P00013');) \| \| --- \| | [Cell cycle (P00013)](http://www.pantherdb.org/list/list.do?chartCategoryAcc=P00013&chartCategoryType=3&filterLevel=1&listType=1) |
| \| [](javascript:popup('colorPallete.jsp?catSelection=P00014');) \| \| --- \| | [Cholesterol biosynthesis (P00014)](http://www.pantherdb.org/list/list.do?chartCategoryAcc=P00014&chartCategoryType=3&filterLevel=1&listType=1) |
| \| [](javascript:popup('colorPallete.jsp?catSelection=P02736');) \| \| --- \| | [Coenzyme A biosynthesis (P02736)](http://www.pantherdb.org/list/list.do?chartCategoryAcc=P02736&chartCategoryType=3&filterLevel=1&listType=1) |
| \| [](javascript:popup('colorPallete.jsp?catSelection=P04380');) \| \| --- \| | [Cortocotropin releasing factor receptor signaling pathway (P04380)](http://www.pantherdb.org/list/list.do?chartCategoryAcc=P04380&chartCategoryType=3&filterLevel=1&listType=1) |
| \| [](javascript:popup('colorPallete.jsp?catSelection=P00016');) \| \| --- \| | [Cytoskeletal regulation by Rho GTPase (P00016)](http://www.pantherdb.org/list/list.do?chartCategoryAcc=P00016&chartCategoryType=3&filterLevel=1&listType=1) |
| \| [](javascript:popup('colorPallete.jsp?catSelection=P00017');) \| \| --- \| | [DNA replication (P00017)](http://www.pantherdb.org/list/list.do?chartCategoryAcc=P00017&chartCategoryType=3&filterLevel=1&listType=1) |
| \| [](javascript:popup('colorPallete.jsp?catSelection=P02738');) \| \| --- \| | [De novo purine biosynthesis (P02738)](http://www.pantherdb.org/list/list.do?chartCategoryAcc=P02738&chartCategoryType=3&filterLevel=1&listType=1) |
| \| [](javascript:popup('colorPallete.jsp?catSelection=P02739');) \| \| --- \| | [De novo pyrimidine deoxyribonucleotide biosynthesis (P02739)](http://www.pantherdb.org/list/list.do?chartCategoryAcc=P02739&chartCategoryType=3&filterLevel=1&listType=1) |
| \| [](javascript:popup('colorPallete.jsp?catSelection=P02740');) \| \| --- \| | [De novo pyrimidine ribonucleotides biosythesis (P02740)](http://www.pantherdb.org/list/list.do?chartCategoryAcc=P02740&chartCategoryType=3&filterLevel=1&listType=1) |
| \| [](javascript:popup('colorPallete.jsp?catSelection=P05912');) \| \| --- \| | [Dopamine receptor mediated signaling pathway (P05912)](http://www.pantherdb.org/list/list.do?chartCategoryAcc=P05912&chartCategoryType=3&filterLevel=1&listType=1) |
| \| [](javascript:popup('colorPallete.jsp?catSelection=P00018');) \| \| --- \| | [EGF receptor signaling pathway (P00018)](http://www.pantherdb.org/list/list.do?chartCategoryAcc=P00018&chartCategoryType=3&filterLevel=1&listType=1) |
| \| [](javascript:popup('colorPallete.jsp?catSelection=P05730');) \| \| --- \| | [Endogenous cannabinoid signaling (P05730)](http://www.pantherdb.org/list/list.do?chartCategoryAcc=P05730&chartCategoryType=3&filterLevel=1&listType=1) |
| \| [](javascript:popup('colorPallete.jsp?catSelection=P00019');) \| \| --- \| | [Endothelin signaling pathway (P00019)](http://www.pantherdb.org/list/list.do?chartCategoryAcc=P00019&chartCategoryType=3&filterLevel=1&listType=1) |
| \| [](javascript:popup('colorPallete.jsp?catSelection=P05913');) \| \| --- \| | [Enkephalin release (P05913)](http://www.pantherdb.org/list/list.do?chartCategoryAcc=P05913&chartCategoryType=3&filterLevel=1&listType=1) |
| \| [](javascript:popup('colorPallete.jsp?catSelection=P00020');) \| \| --- \| | [FAS signaling pathway (P00020)](http://www.pantherdb.org/list/list.do?chartCategoryAcc=P00020&chartCategoryType=3&filterLevel=1&listType=1) |
| \| [](javascript:popup('colorPallete.jsp?catSelection=P00021');) \| \| --- \| | [FGF signaling pathway (P00021)](http://www.pantherdb.org/list/list.do?chartCategoryAcc=P00021&chartCategoryType=3&filterLevel=1&listType=1) |
| \| [](javascript:popup('colorPallete.jsp?catSelection=P02743');) \| \| --- \| | [Formyltetrahydroformate biosynthesis (P02743)](http://www.pantherdb.org/list/list.do?chartCategoryAcc=P02743&chartCategoryType=3&filterLevel=1&listType=1) |
| \| [](javascript:popup('colorPallete.jsp?catSelection=P02744');) \| \| --- \| | [Fructose galactose metabolism (P02744)](http://www.pantherdb.org/list/list.do?chartCategoryAcc=P02744&chartCategoryType=3&filterLevel=1&listType=1) |
| \| [](javascript:popup('colorPallete.jsp?catSelection=P05731');) \| \| --- \| | [GABA-B receptor II signaling (P05731)](http://www.pantherdb.org/list/list.do?chartCategoryAcc=P05731&chartCategoryType=3&filterLevel=1&listType=1) |
| \| [](javascript:popup('colorPallete.jsp?catSelection=P04384');) \| \| --- \| | [Gamma-aminobutyric acid synthesis (P04384)](http://www.pantherdb.org/list/list.do?chartCategoryAcc=P04384&chartCategoryType=3&filterLevel=1&listType=1) |
| \| [](javascript:popup('colorPallete.jsp?catSelection=P00022');) \| \| --- \| | [General transcription by RNA polymerase I (P00022)](http://www.pantherdb.org/list/list.do?chartCategoryAcc=P00022&chartCategoryType=3&filterLevel=1&listType=1) |
| \| [](javascript:popup('colorPallete.jsp?catSelection=P00023');) \| \| --- \| | [General transcription regulation (P00023)](http://www.pantherdb.org/list/list.do?chartCategoryAcc=P00023&chartCategoryType=3&filterLevel=1&listType=1) |
| \| [](javascript:popup('colorPallete.jsp?catSelection=P00024');) \| \| --- \| | [Glycolysis (P00024)](http://www.pantherdb.org/list/list.do?chartCategoryAcc=P00024&chartCategoryType=3&filterLevel=1&listType=1) |
| \| [](javascript:popup('colorPallete.jsp?catSelection=P06664');) \| \| --- \| | [Gonadotropin-releasing hormone receptor pathway (P06664)](http://www.pantherdb.org/list/list.do?chartCategoryAcc=P06664&chartCategoryType=3&filterLevel=1&listType=1) |
| \| [](javascript:popup('colorPallete.jsp?catSelection=P00025');) \| \| --- \| | [Hedgehog signaling pathway (P00025)](http://www.pantherdb.org/list/list.do?chartCategoryAcc=P00025&chartCategoryType=3&filterLevel=1&listType=1) |
| \| [](javascript:popup('colorPallete.jsp?catSelection=P02746');) \| \| --- \| | [Heme biosynthesis (P02746)](http://www.pantherdb.org/list/list.do?chartCategoryAcc=P02746&chartCategoryType=3&filterLevel=1&listType=1) |
| \| [](javascript:popup('colorPallete.jsp?catSelection=P00026');) \| \| --- \| | [Heterotrimeric G-protein signaling pathway-Gi alpha and Gs alpha mediated pathway (P00026)](http://www.pantherdb.org/list/list.do?chartCategoryAcc=P00026&chartCategoryType=3&filterLevel=1&listType=1) |
| \| [](javascript:popup('colorPallete.jsp?catSelection=P00027');) \| \| --- \| | [Heterotrimeric G-protein signaling pathway-Gq alpha and Go alpha mediated pathway (P00027)](http://www.pantherdb.org/list/list.do?chartCategoryAcc=P00027&chartCategoryType=3&filterLevel=1&listType=1) |
| \| [](javascript:popup('colorPallete.jsp?catSelection=P00028');) \| \| --- \| | [Heterotrimeric G-protein signaling pathway-rod outer segment phototransduction (P00028)](http://www.pantherdb.org/list/list.do?chartCategoryAcc=P00028&chartCategoryType=3&filterLevel=1&listType=1) |
| \| [](javascript:popup('colorPallete.jsp?catSelection=P04385');) \| \| --- \| | [Histamine H1 receptor mediated signaling pathway (P04385)](http://www.pantherdb.org/list/list.do?chartCategoryAcc=P04385&chartCategoryType=3&filterLevel=1&listType=1) |
| \| [](javascript:popup('colorPallete.jsp?catSelection=P04386');) \| \| --- \| | [Histamine H2 receptor mediated signaling pathway (P04386)](http://www.pantherdb.org/list/list.do?chartCategoryAcc=P04386&chartCategoryType=3&filterLevel=1&listType=1) |
| \| [](javascript:popup('colorPallete.jsp?catSelection=P00029');) \| \| --- \| | [Huntington disease (P00029)](http://www.pantherdb.org/list/list.do?chartCategoryAcc=P00029&chartCategoryType=3&filterLevel=1&listType=1) |
| \| [](javascript:popup('colorPallete.jsp?catSelection=P00030');) \| \| --- \| | [Hypoxia response via HIF activation (P00030)](http://www.pantherdb.org/list/list.do?chartCategoryAcc=P00030&chartCategoryType=3&filterLevel=1&listType=1) |
| \| [](javascript:popup('colorPallete.jsp?catSelection=P00031');) \| \| --- \| | [Inflammation mediated by chemokine and cytokine signaling pathway (P00031)](http://www.pantherdb.org/list/list.do?chartCategoryAcc=P00031&chartCategoryType=3&filterLevel=1&listType=1) |
| \| [](javascript:popup('colorPallete.jsp?catSelection=P00032');) \| \| --- \| | [Insulin/IGF pathway-mitogen activated protein kinase kinase/MAP kinase cascade (P00032)](http://www.pantherdb.org/list/list.do?chartCategoryAcc=P00032&chartCategoryType=3&filterLevel=1&listType=1) |
| \| [](javascript:popup('colorPallete.jsp?catSelection=P00033');) \| \| --- \| | [Insulin/IGF pathway-protein kinase B signaling cascade (P00033)](http://www.pantherdb.org/list/list.do?chartCategoryAcc=P00033&chartCategoryType=3&filterLevel=1&listType=1) |
| \| [](javascript:popup('colorPallete.jsp?catSelection=P00034');) \| \| --- \| | [Integrin signalling pathway (P00034)](http://www.pantherdb.org/list/list.do?chartCategoryAcc=P00034&chartCategoryType=3&filterLevel=1&listType=1) |
| \| [](javascript:popup('colorPallete.jsp?catSelection=P00035');) \| \| --- \| | [Interferon-gamma signaling pathway (P00035)](http://www.pantherdb.org/list/list.do?chartCategoryAcc=P00035&chartCategoryType=3&filterLevel=1&listType=1) |
| \| [](javascript:popup('colorPallete.jsp?catSelection=P00036');) \| \| --- \| | [Interleukin signaling pathway (P00036)](http://www.pantherdb.org/list/list.do?chartCategoryAcc=P00036&chartCategoryType=3&filterLevel=1&listType=1) |
| \| [](javascript:popup('colorPallete.jsp?catSelection=P00037');) \| \| --- \| | [Ionotropic glutamate receptor pathway (P00037)](http://www.pantherdb.org/list/list.do?chartCategoryAcc=P00037&chartCategoryType=3&filterLevel=1&listType=1) |
| \| [](javascript:popup('colorPallete.jsp?catSelection=P00038');) \| \| --- \| | [JAK/STAT signaling pathway (P00038)](http://www.pantherdb.org/list/list.do?chartCategoryAcc=P00038&chartCategoryType=3&filterLevel=1&listType=1) |
| \| [](javascript:popup('colorPallete.jsp?catSelection=P00041');) \| \| --- \| | [Metabotropic glutamate receptor group I pathway (P00041)](http://www.pantherdb.org/list/list.do?chartCategoryAcc=P00041&chartCategoryType=3&filterLevel=1&listType=1) |
| \| [](javascript:popup('colorPallete.jsp?catSelection=P00040');) \| \| --- \| | [Metabotropic glutamate receptor group II pathway (P00040)](http://www.pantherdb.org/list/list.do?chartCategoryAcc=P00040&chartCategoryType=3&filterLevel=1&listType=1) |
| \| [](javascript:popup('colorPallete.jsp?catSelection=P00039');) \| \| --- \| | [Metabotropic glutamate receptor group III pathway (P00039)](http://www.pantherdb.org/list/list.do?chartCategoryAcc=P00039&chartCategoryType=3&filterLevel=1&listType=1) |
| \| [](javascript:popup('colorPallete.jsp?catSelection=P00042');) \| \| --- \| | [Muscarinic acetylcholine receptor 1 and 3 signaling pathway (P00042)](http://www.pantherdb.org/list/list.do?chartCategoryAcc=P00042&chartCategoryType=3&filterLevel=1&listType=1) |
| \|  \| \| --- \| | [Muscarinic acetylcholine receptor 2 and 4 signaling pathway (P00043)](http://www.pantherdb.org/list/list.do?chartCategoryAcc=P00043&chartCategoryType=3&filterLevel=1&listType=1) |
| \|  \| \| --- \| | [Nicotine pharmacodynamics pathway (P06587)](http://www.pantherdb.org/list/list.do?chartCategoryAcc=P06587&chartCategoryType=3&filterLevel=1&listType=1) |
| \|  \| \| --- \| | [Nicotinic acetylcholine receptor signaling pathway (P00044)](http://www.pantherdb.org/list/list.do?chartCategoryAcc=P00044&chartCategoryType=3&filterLevel=1&listType=1) |
| \|  \| \| --- \| | [Notch signaling pathway (P00045)](http://www.pantherdb.org/list/list.do?chartCategoryAcc=P00045&chartCategoryType=3&filterLevel=1&listType=1) |
| \|  \| \| --- \| | [Opioid prodynorphin pathway (P05916)](http://www.pantherdb.org/list/list.do?chartCategoryAcc=P05916&chartCategoryType=3&filterLevel=1&listType=1) |
| \|  \| \| --- \| | [Opioid proenkephalin pathway (P05915)](http://www.pantherdb.org/list/list.do?chartCategoryAcc=P05915&chartCategoryType=3&filterLevel=1&listType=1) |
| \|  \| \| --- \| | [Opioid proopiomelanocortin pathway (P05917)](http://www.pantherdb.org/list/list.do?chartCategoryAcc=P05917&chartCategoryType=3&filterLevel=1&listType=1) |
| \|  \| \| --- \| | [Ornithine degradation (P02758)](http://www.pantherdb.org/list/list.do?chartCategoryAcc=P02758&chartCategoryType=3&filterLevel=1&listType=1) |
| \|  \| \| --- \| | [Oxidative stress response (P00046)](http://www.pantherdb.org/list/list.do?chartCategoryAcc=P00046&chartCategoryType=3&filterLevel=1&listType=1) |
| \|  \| \| --- \| | [Oxytocin receptor mediated signaling pathway (P04391)](http://www.pantherdb.org/list/list.do?chartCategoryAcc=P04391&chartCategoryType=3&filterLevel=1&listType=1) |
| \|  \| \| --- \| | [P53 pathway feedback loops 1 (P04392)](http://www.pantherdb.org/list/list.do?chartCategoryAcc=P04392&chartCategoryType=3&filterLevel=1&listType=1) |
| \|  \| \| --- \| | [PDGF signaling pathway (P00047)](http://www.pantherdb.org/list/list.do?chartCategoryAcc=P00047&chartCategoryType=3&filterLevel=1&listType=1) |
| \|  \| \| --- \| | [PI3 kinase pathway (P00048)](http://www.pantherdb.org/list/list.do?chartCategoryAcc=P00048&chartCategoryType=3&filterLevel=1&listType=1) |
| \|  \| \| --- \| | [Parkinson disease (P00049)](http://www.pantherdb.org/list/list.do?chartCategoryAcc=P00049&chartCategoryType=3&filterLevel=1&listType=1) |
| \|  \| \| --- \| | [Pentose phosphate pathway (P02762)](http://www.pantherdb.org/list/list.do?chartCategoryAcc=P02762&chartCategoryType=3&filterLevel=1&listType=1) |
| \|  \| \| --- \| | [Phenylethylamine degradation (P02766)](http://www.pantherdb.org/list/list.do?chartCategoryAcc=P02766&chartCategoryType=3&filterLevel=1&listType=1) |
| \|  \| \| --- \| | [Purine metabolism (P02769)](http://www.pantherdb.org/list/list.do?chartCategoryAcc=P02769&chartCategoryType=3&filterLevel=1&listType=1) |
| \|  \| \| --- \| | [Pyridoxal-5-phosphate biosynthesis (P02759)](http://www.pantherdb.org/list/list.do?chartCategoryAcc=P02759&chartCategoryType=3&filterLevel=1&listType=1) |
| \|  \| \| --- \| | [Pyrimidine Metabolism (P02771)](http://www.pantherdb.org/list/list.do?chartCategoryAcc=P02771&chartCategoryType=3&filterLevel=1&listType=1) |
| \|  \| \| --- \| | [Pyruvate metabolism (P02772)](http://www.pantherdb.org/list/list.do?chartCategoryAcc=P02772&chartCategoryType=3&filterLevel=1&listType=1) |
| \|  \| \| --- \| | [Ras Pathway (P04393)](http://www.pantherdb.org/list/list.do?chartCategoryAcc=P04393&chartCategoryType=3&filterLevel=1&listType=1) |
| \|  \| \| --- \| | [Salvage pyrimidine ribonucleotides (P02775)](http://www.pantherdb.org/list/list.do?chartCategoryAcc=P02775&chartCategoryType=3&filterLevel=1&listType=1) |
| \|  \| \| --- \| | [Serine glycine biosynthesis (P02776)](http://www.pantherdb.org/list/list.do?chartCategoryAcc=P02776&chartCategoryType=3&filterLevel=1&listType=1) |
| \|  \| \| --- \| | [Synaptic vesicle trafficking (P05734)](http://www.pantherdb.org/list/list.do?chartCategoryAcc=P05734&chartCategoryType=3&filterLevel=1&listType=1) |
| \|  \| \| --- \| | [T cell activation (P00053)](http://www.pantherdb.org/list/list.do?chartCategoryAcc=P00053&chartCategoryType=3&filterLevel=1&listType=1) |
| \|  \| \| --- \| | [TCA cycle (P00051)](http://www.pantherdb.org/list/list.do?chartCategoryAcc=P00051&chartCategoryType=3&filterLevel=1&listType=1) |
| \|  \| \| --- \| | [TGF-beta signaling pathway (P00052)](http://www.pantherdb.org/list/list.do?chartCategoryAcc=P00052&chartCategoryType=3&filterLevel=1&listType=1) |
| \|  \| \| --- \| | [Tetrahydrofolate biosynthesis (P02742)](http://www.pantherdb.org/list/list.do?chartCategoryAcc=P02742&chartCategoryType=3&filterLevel=1&listType=1) |
| \|  \| \| --- \| | [Thiamin metabolism (P02780)](http://www.pantherdb.org/list/list.do?chartCategoryAcc=P02780&chartCategoryType=3&filterLevel=1&listType=1) |
| \|  \| \| --- \| | [Threonine biosynthesis (P02781)](http://www.pantherdb.org/list/list.do?chartCategoryAcc=P02781&chartCategoryType=3&filterLevel=1&listType=1) |
| \|  \| \| --- \| | [Thyrotropin-releasing hormone receptor signaling pathway (P04394)](http://www.pantherdb.org/list/list.do?chartCategoryAcc=P04394&chartCategoryType=3&filterLevel=1&listType=1) |
| \|  \| \| --- \| | [Toll receptor signaling pathway (P00054)](http://www.pantherdb.org/list/list.do?chartCategoryAcc=P00054&chartCategoryType=3&filterLevel=1&listType=1) |
| \|  \| \| --- \| | [Transcription regulation by bZIP transcription factor (P00055)](http://www.pantherdb.org/list/list.do?chartCategoryAcc=P00055&chartCategoryType=3&filterLevel=1&listType=1) |
| \|  \| \| --- \| | [Ubiquitin proteasome pathway (P00060)](http://www.pantherdb.org/list/list.do?chartCategoryAcc=P00060&chartCategoryType=3&filterLevel=1&listType=1) |
| \|  \| \| --- \| | [VEGF signaling pathway (P00056)](http://www.pantherdb.org/list/list.do?chartCategoryAcc=P00056&chartCategoryType=3&filterLevel=1&listType=1) |
| \|  \| \| --- \| | [Vitamin B6 metabolism (P02787)](http://www.pantherdb.org/list/list.do?chartCategoryAcc=P02787&chartCategoryType=3&filterLevel=1&listType=1) |
| \|  \| \| --- \| | [Vitamin D metabolism and pathway (P04396)](http://www.pantherdb.org/list/list.do?chartCategoryAcc=P04396&chartCategoryType=3&filterLevel=1&listType=1) |
| \|  \| \| --- \| | [Wnt signaling pathway (P00057)](http://www.pantherdb.org/list/list.do?chartCategoryAcc=P00057&chartCategoryType=3&filterLevel=1&listType=1) |
| \|  \| \| --- \| | [Xanthine and guanine salvage pathway (P02788)](http://www.pantherdb.org/list/list.do?chartCategoryAcc=P02788&chartCategoryType=3&filterLevel=1&listType=1) |
| \|  \| \| --- \| | [mRNA splicing (P00058)](http://www.pantherdb.org/list/list.do?chartCategoryAcc=P00058&chartCategoryType=3&filterLevel=1&listType=1) |
| \|  \| \| --- \| | [p38 MAPK pathway (P05918)](http://www.pantherdb.org/list/list.do?chartCategoryAcc=P05918&chartCategoryType=3&filterLevel=1&listType=1) |
| \|  \| \| --- \| | [p53 pathway by glucose deprivation (P04397)](http://www.pantherdb.org/list/list.do?chartCategoryAcc=P04397&chartCategoryType=3&filterLevel=1&listType=1) |
| \|  \| \| --- \| | [p53 pathway feedback loops 2 (P04398)](http://www.pantherdb.org/list/list.do?chartCategoryAcc=P04398&chartCategoryType=3&filterLevel=1&listType=1) |
| \|  \| \| --- \| | [p53 pathway (P00059)](http://www.pantherdb.org/list/list.do?chartCategoryAcc=P00059&chartCategoryType=3&filterLevel=1&listType=1) |

**Figure S2.** **Classification into pathways of the dysregulated genes in LF82-infected *versus* uninfected M cells that were potentially regulated by miRNAs.** The GENEONTOLOGY PANTHER (Protein ANalysis THrough Evolutionary Relationships) Classification System was used:

(<http://www.pantherdb.org/chart/pantherChart.jsp?listType=1&filterLevel=1&type=3&chartType=2&save=yes&basketItems=all&zoom=1&trackingId=042BE96F5118881E0C45A6320456E8B4>).

**Table S1.** **Dysregulated miRNAs in uninfected M cells compared to uninfected Caco-2-cl1 cells.** A total of 10^7^ LF82 bacteria were added into the apical compartment of M cells or the Caco-2-cl1 monolayers. At 4 h post-infection, the monolayers were collected, washed with cold PBS, and total RNA was extracted and used for miRNA array analysis. The miRNAs that were dysregulated in uninfected M cells compared to uninfected Caco-2-cl1 cells were presented. The upregulated miRNAs are shown in red and the downregulated miRNAs are shown in green.

**Table S2.** **Potential target genes of dysregulated miRNAs in uninfected M cells compared to uninfected Caco-2-cl1 cells.** A total of 10^7^ LF82 bacteria were added into the apical compartment of the M-cell or the Caco-2-cl1 monolayers. At 4 h post-infection, the monolayers were collected, washed with cold PBS, and total RNA was extracted and used for miRNA array analysis. The potential target genes of dysregulated miRNAs (upregulated miRNAs in red and downregulated miRNAs in green) in uninfected M cells vs. Caco-2-cl1 cells were predicted by in silico analysis using the MirWalk (version 3.0) database.

**Table S3. Dysregulated genes in uninfected M cells versus uninfected Caco-2-cl1 cells.** A total of 10^7^ LF82 bacteria were added into the apical compartment of the M cells or the Caco-2-cl1 monolayers. At 4 h post-infection, the monolayers were collected, washed with cold PBS, and total RNA was extracted and used for microarray analysis. The genes that were dysregulated in uninfected M cells compared to uninfected Caco-2-cl1 cells were presented. The upregulated genes are shown in red and the downregulated genes are shown in green.

**Table S4**. **Dysregulated genes in uninfected M cells compared to Caco-2-cl1 cells that were potentially regulated by miRNAs.** The downregulated genes in uninfected M cells vs. uninfected Caco-2-cl1 cells identified by microarray analysis were matched with the potential target genes of upregulated miRNAs in uninfected M cells vs. uninfected Caco-2-cl1 cells revealed by in silico analysis. The upregulated genes in uninfected M cells vs. uninfected Caco-2-cl1 cells identified by microarray analysis were matched with the potential target genes of downregulated miRNAs in uninfected M cells vs. uninfected Caco-2-cl1 cells revealed by in silico analysis. The matched genes were presented and were considered as dysregulated genes in uninfected M cells compared to Caco-2-cl1 cells that were potentially regulated by miRNAs.

**Table S5.** **Dysregulated miRNAs in AIEC LF82-infected versus uninfected Caco-2-cl1 cells.** A total of 10^7^ LF82 bacteria were added into the apical compartment of Caco-2-cl1 monolayers. At 4 h post-infection, the monolayers were collected, washed with cold PBS, and total RNA was extracted and used for miRNA array analysis. The miRNAs that were dysregulated in AIEC LF82-infected vs. uninfected Caco-2-cl1 cells were presented. The upregulated miRNAs are shown in red and the downregulated miRNAs are shown in green.

**Table S6.** **Dysregulated miRNAs in AIEC LF82-infected versus uninfected M cells.** A total of 10^7^ LF82 bacteria were added into the apical compartment of M cells. At 4 h post-infection, the monolayers were collected, washed with cold PBS, and total RNA was extracted and used for miRNA array analysis. The miRNAs that were dysregulated in AIEC LF82-infected vs. uninfected M cells were presented. The upregulated miRNAs are shown in red and the downregulated miRNAs are shown in green.

**Table S7.** **Potential target genes of dysregulated miRNAs in LF82-infected compared to uninfected Caco-2-cl1 cells.** Caco-2-cl1 monolayers uninfected or infected with 10^7^ LF82 bacteria for 4 h were collected, washed with cold PBS, and total RNA was extracted and used for miRNA array analysis. The potential target genes of dysregulated miRNAs (upregulated miRNAs in red and downregulated miRNAs in green) in LF82-infected vs. uninfected Caco-2-cl1 cells were predicted by in silico analysis using the MirWalk (version 3.0) database.

**Table S8**. **Potential target genes of dysregulated miRNAs in LF82-infected compared to uninfected M cells.** M cells uninfected or infected with 10^7^ LF82 bacteria for 4 h were collected, washed with cold PBS, and total RNA was extracted and used for miRNA array analysis. The potential target genes of dysregulated miRNAs (upregulated miRNAs in red and downregulated miRNAs in green) in LF82-infected vs. uninfected M cells were predicted by in silico analysis using the MirWalk (version 3.0) database.

**Table S9.** **Dysregulated genes in AIEC LF82-infected versus uninfected Caco-2-cl1 cells.** A total of 10^7^ LF82 bacteria were added into the apical compartment of the Caco-2-cl1 monolayers. At 4 h post-infection, the monolayers were collected, washed with cold PBS, and total RNA was extracted and used for microarray analysis. The genes that were dysregulated in AIEC LF82-infected vs. uninfected Caco-2-cl1 cells were presented. The upregulated genes were shown in red and the downregulated genes were shown in green.

**Table S10**. **Dysregulated genes in AIEC LF82-infected versus uninfected M cells.** A total of 10^7^ LF82 bacteria were added into the apical compartment of the M cells. At 4 h post-infection, the monolayers were collected, washed with cold PBS, and total RNA was extracted and used for microarray analysis. The genes that were dysregulated in AIEC LF82-infected vs. uninfected M cells were presented. The upregulated genes are shown in red and the downregulated genes are shown in green.

**Table S11.** **Genes that were dysregulated in both Caco-2-cl1 cells and M cells upon AIEC LF82 infection.** A total of 10^7^ LF82 bacteria were added into the apical compartment of the M cell or the Caco-2-cl1 monolayers. At 4 h post-infection, the monolayers were collected, washed with cold PBS, and total RNA was extracted and used for miRNA array analysis. The miRNAs that were dysregulated in uninfected M cells compared to uninfected Caco-2-cl1 cells were presented. The upregulated miRNAs are shown in red and the downregulated miRNAs are shown in green.

**Table S12.** **Dysregulated genes in LF82-infected versus uninfected Caco-2-cl1 cells that were potentially regulated by miRNAs.** The downregulated genes in LF82-infected vs. uninfected Caco-2-cl1 cells identified by microarray analysis were matched with the potential target genes of upregulated miRNAs in LF82-infected vs. uninfected Caco-2-cl1 cells revealed by in silico analysis. The upregulated genes in LF82-infected vs. uninfected Caco-2-cl1 cells identified by microarray analysis were matched with the potential target genes of downregulated miRNAs in LF82-infected vs. uninfected Caco-2-cl1 cells revealed by in silico analysis. The matched genes were presented and were considered as dysregulated genes in LF82-infected vs. uninfected Caco-2-cl1 cells that were potentially regulated by miRNAs.

**Table S13.** **Dysregulated genes in LF82-infected versus uninfected M cells that were potentially regulated by miRNAs.** The downregulated genes in LF82-infected vs. uninfected M cells identified by microarray analysis were matched with the potential target genes of upregulated miRNAs in LF82-infected vs. uninfected M cells revealed by in silico analysis. The upregulated genes in LF82-infected vs. uninfected M cells identified by microarray analysis were matched with the potential target genes of downregulated miRNAs in LF82-infected vs. uninfected M cells revealed by in silico analysis. The matched genes were presented and were considered as dysregulated genes in LF82-infected vs. uninfected M cells that were potentially regulated by miRNAs.

**Table S14. Genes that were dysregulated in both Caco-2-cl1 cells and M cells upon AIEC LF82 infection and were potentially targeted by miRNAs**. Comparison between the dysregulated genes potentially targeted by the dysregulated miRNAs upon AIEC infection of Caco-2-cl1 cells and of M cells revealed that LF82-induced dysregulated miRNAs potentially target 48 downregulated and 36 upregulated genes in both cell lines.
